# Hit‐To‐Lead Optimization of a Pyridylpiperazine Class Against Malaria: Pharmacokinetic Profile and In Vivo Efficacy of Optimized Compounds

**DOI:** 10.1002/cmdc.70373

**Published:** 2026-07-08

**Authors:** Douglas Davison da Silva Oliveira, Duarte Eduardo Pereira, Rafael Consolin Chelucci, Simone Michelan, Júlia Maria Fernandes Pituba, Adriano D. Andricopulo, Leonardo L. G. Ferreira, Neide Maria Silva, Celso de Oliveira Rezende Júnior

**Affiliations:** ^1^ Laboratório de Síntese de Candidatos a Fármacos (LaSFar) Institute of Chemistry Federal University of Uberlândia (UFU) Uberlândia MG Brazil; ^2^ Laboratory of Immunopathology Institute of Biomedical Sciences Federal University of Uberlândia (UFU) Uberlândia MG Brazil; ^3^ Laboratório de Química Medicinal e Computacional (LQMC) Instituto de Física de São Carlos (IFSC) Universidade de São Paulo (USP) São Carlos SP Brazil; ^4^ Departamento de Química Universidade Federal de Juiz de Fora (UFJF) Juiz de Fora MG Brazil

**Keywords:** ADME, antimalarial activity, drug discovery, parasitic disease, proof of concept

## Abstract

Malaria remains a significant global health challenge caused by *Plasmodium* parasites. Current antimalarial treatment strategies face mounting challenges due to the rapid development of drug resistance. This work aimed at the hit‐to‐lead optimization of a pyridylpiperazine class by evaluating the in vitro pharmacokinetic profile and the in vivo efficacy and safety against malaria. The systematic in vitro assessment of ADME properties for 10 previously selected bioactive compounds enabled a detailed structure–ADME relationship analysis. This process highlighted compounds **7** and **9** as having the most promising profiles for improved bioavailability. These optimized compounds were then tested in an in vivo murine model of malaria, and reduced the likelihood of mice developing cerebral malaria. Compound **7** emerged as the most effective, reducing parasitemia by up to 91.7%. These results successfully validate that chemical modifications to the toluyl fragment of the initial hit optimized the pharmacodynamic and pharmacokinetic parameters, yielding two candidates with suitable ADME profiles and proven in vivo efficacy against malaria. This work supports the further development of the pyridylpiperazine class against malaria and highlights the need to explore modifications on other parts of the molecular scaffold and elucidate their mechanism of action.

## Graphical abstract

Chemical optimization of a novel pyridylpiperazine class yields two promising antimalarial candidates with an exceptional balance of metabolic stability and solubility. These optimized leads significantly protect mice from cerebral malaria, reducing parasitic load by up to 91.7%. This study validates a strategic approach to overcoming drug resistance, paving the way for advanced preclinical development.

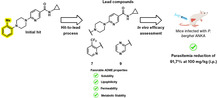

## 1 Introduction

Malaria remains a significant global health challenge, with an estimated 263 million cases and 597,000 deaths reported in 2023, affecting 83 countries worldwide. The African continent is the most affected, accounting for approximately 94% of all malaria cases and 95% of malaria‐related deaths, with children under 5 years old representing 76% of fatalities [1, 2]. Malaria is a disease caused by parasites belonging to the genus *Plasmodium* (*P.*) and transmitted by infected female *Anopheles* mosquitoes. There are five species of *Plasmodium* responsible for infecting humans: *P. falciparum*, *P. malariae*, *P. vivax*, *P.* *ovale*, *and P. knowlesi*. Among these species, *P. falciparum* and *P. vivax* are responsible for the largest number of cases and deaths, with *P. falciparum* being the most lethal and prevalent in Africa, while *P. vivax* dominates outside sub‐Saharan Africa [1, 3, 4].

The life cycle of *Plasmodium* includes multiple developmental stages in both mosquito vectors and human hosts. Upon transmission through a mosquito bite, sporozoites travel to the liver, where they mature into merozoites before infecting red blood cells (RBCs), leading to clinical symptoms. While mild cases present as fever, chills, and headaches, severe infections can result in complications such as seizures, organ failure, and death. Certain populations, including pregnant women, young children, immunocompromised individuals, and travelers to endemic regions, are at higher risk of severe disease [1, 5].

Current malaria control strategies focus on vector management, preventive chemotherapies, and treatment regimens. Insecticide‐treated nets (ITNs) and indoor residual spraying (IRS) are core interventions to reduce mosquito populations and transmission rates. However, resistance to insecticides among *Anopheles* mosquitoes, particularly the invasive *Anopheles stephensi* species, threatens these efforts. Chemoprophylaxis remains essential for travelers to endemic regions, and preventive chemotherapy is implemented for at‐risk populations, including pregnant women and children. The RTS,S/AS01 vaccine, recommended by WHO since 2021, and the recently approved R21/Matrix‐M vaccine represent critical advancements in malaria prevention, with ongoing rollout in Africa expected to significantly reduce mortality rates when combined with existing interventions. Nevertheless, both vaccines confer only partial and time‐limited protection, with moderate efficacy that declines over time and varies according to transmission settings. Therefore, their implementation is intended to complement existing control measures, rather than serve as standalone solutions, reinforcing the need for continued development of new therapeutic and preventive strategies [6, 7, 8].

Despite these efforts, malaria treatment faces growing challenges due to antimalarial drug resistance. Artemisinin‐based combination therapies (ACTs) remain the most effective treatment for *P. falciparum* malaria, while chloroquine is used in areas where *P. vivax* remains susceptible. However, resistance to artemisinin has been confirmed in parts of Africa, including Eritrea, Rwanda, Uganda, and Tanzania, raising concerns about treatment efficacy [9, 10]. The WHO has implemented strategies to monitor and address drug resistance, emphasizing the need for continuous surveillance and novel therapeutic approaches [1].

In our previous study, we initiated the optimization of a series of pyridylpiperazine derivatives against *P. falciparum*. This effort was based on the identification of compound **1**, a pyridylpiperazine derivative, which exhibited in vitro potency (IC_50_ *Pf*3D7 = 1.0 μM and IC_50_ *Pf*Dd2 = 0.7 μM) against chloroquine‐sensitive and chloroquine‐resistant strains, respectively (Table 1). Our structure–activity relationship (SAR) study focused on the toluyl fragment of compound **1**, hypothesized to be particularly susceptible to oxidative metabolism due to its toluyl group [11]. This previous work led to the identification of nine derivatives with IC_50_  values below 6 μM against *P. falciparum*, establishing them as promising lead candidates for further development. Additionally, there is no evidence regarding the mechanism of action of these compounds. While the SAR study provided critical insights into the structural features that govern antiplasmodial activity, it also highlighted the need for subsequent investigations into the pharmacokinetic properties and in vivo efficacy of these optimized derivatives. To address this gap, the present study significantly advances the hit‐to‐lead optimization of the pyridylpiperazine class, detailing the comprehensive results from in vitro ADME profiling and in vivo efficacy assessment in a murine model of malaria.

**TABLE 1:** Phenotypic and ADME profiling of the compounds **1–10**.

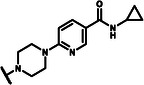												
Comp.	Structure	IC_50_ *Pf*3D7 (μM)^a^	IC_50_ *Pf*Dd2 (μM)^b^	CC_50_ HepG2 (μM)^c^	PAMPA (10^−6^ cm/s)^d^	KS pH 2.0 (μM)^e^	KS pH 7.4 (μM)^e^	eLogD pH 7.4^f^	MLM CL_int_ (μL/min/mg)^g^	MLM *t*_1/2_ (min)^h^	HLM CL_int_ (μL/min/mg)^i^	HLM *t*_1/2_ (min)^j^
1	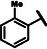	1.0	0.7	>100	15.96	>81	>84	4.07	38.80	71.46	44.4	62.45
2	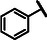	4.7	3.3	>100	20.41	>65	26.02	3.36	20.40	135.91	218.40	12.70
3	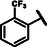	1.7	0.5	>100	3.28	>68	>64	4.34	100.80	27.51	26.80	103.45
4	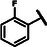	1.3	2.3	315	20.60	138.32	147.14	3.55	37.20	74.53	88.0	31.5
5	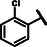	0.4	0.7	6.1	15.54	>132	43.48	4.03	48.40	57.28	30.0	92.42
6	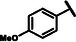	2.9	9.4	46.7	14.86	>67	>42	3.24	35.60	77.88	132.40	20.94
7	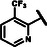	1.0	4.4	>100	12.95	>63	>50	3.83	69.60	39.84	31.20	88.87
8	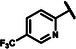	1.1	1.6	16.2	7.44	>57	8.32	3.92	42.40	65.39	25.60	108.30
9	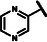	2.8	3.6	>100	8.88	>83	>87	2.35	<12	630.13	25.20	110.02
10	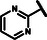	5.6	10.5	22	18.87	>47	>52	2.58	13.60	203.87	27.60	100.46

a IC_50_ values against *P. falciparum* 3D7 (chloroquine‐sensitive strain).

b IC_50_ values against *P. falciparum* Dd2 (chloroquine‐resistant strain).

c Cytotoxicity (CC_50_) values in HepG2 cells.

d Permeability coefficients determined by PAMPA assay.

e Kinetic solubility at pH 2.0 and 7.4.

f Lipophilicity values expressed as eLogD at pH 7.4.

g Metabolic stability expressed as intrinsic clearance (CL_int_) in mouse liver microsomes (MLM).

h Plasma half‐life (*t*_1/2_) in MLM.

i Metabolic stability expressed as intrinsic clearance (CL_int_) in human liver microsomes (HLM).

j Plasma half‐life (*t*_1/2_) HLM. The tests were performed in triplicates. Positive controls used: PAMPA (nadolol and metoprolol); kinetic solubility (amiodarone, diclofenac, chloramphenicol, and itraconazole); eLogD (acyclovir, atenolol, antipyrine, fluconazole, metoprolol, ketoconazole, tolnaftate, and amiodarone); metabolic stability (imipramine, verapamil, and warfarin).

## 2 Results and Discussion

### 2.1 Selection Criteria for ADME Profiling

The compounds chosen for in vitro ADME profile evaluation were those demonstrating antimalarial activity against both chloroquine‐sensitive (*P. falciparum* 3D7) and chloroquine‐resistant (*P. falciparum* Dd2) strains, specifically those with IC_50_ values less than 6 μM against *P. falciparum* 3D7 strain (Compounds **1–10**, Table 1). Crucially, the majority of these compounds displayed low cytotoxicity (CC_50_ values greater than 100 μM) against HepG2 cells, confirming their selective action against the parasite.

### 2.2 In Vitro ADME Profile

To narrow down the field and identify the most promising candidates for subsequent in vivo assessment in mouse models, the ADME profile was determined in vitro. This complementary step aimed to ensure that the selected compounds possessed favorable pharmacokinetic properties in addition to high efficacy.

Lipophilicity values, measured as eLogD, ranged from 2.35 to 4.34, indicating favorable characteristics for drug‐like behavior (Table 1) [12, 13]. Most of the compounds exhibited eLogD higher than 3. However, compounds **9** and **10**, which are pyrazine and pyrimidine derivatives, respectively, stood out by presenting adequate eLogD values (1 < eLogD < 3) [14]. The reduced lipophilicity of these two compounds was expected, as they are the only derivatives in the series featuring two endocyclic nitrogens in the aryl fragment. Regarding substitutions, replacing the methyl group of the initial hit **1** with hydrogen (**2**), fluorine (**4**), or methoxyl (**6**) consistently led to a reduction in lipophilicity. Conversely, replacement with a trifluoromethyl group (**3**) resulted in the anticipated increase [15]. Furthermore, compounds **7** and **8**, containing the trifluoromethylpyridine fragment, also showed an eLogD value lower than that of the initial hit **1**.

All compounds exhibited favorable permeability, as evidenced by their high gastrointestinal permeability in parallel artificial membrane permeability assays (PAMPA) (Pe > 3.28 µcm/s) [16]. The trifluoromethylbenzene derivative **3** exhibited the lowest permeability (Pe = 3.28 µcm/s) while the fluorobenzene derivative **4** was the most permeable (Pe = 20.60 µcm/s).

Kinetic solubility was also evaluated at pH 2.0 and 7.4, simulating distinct physiological environments. All compounds demonstrated high solubility (KS > 40 µM) at both pH levels [17, 18], except for compounds **2** and **8**, which displayed moderate solubility at pH 7.4 (KS = 26.02 and 8.3 µM, respectively). Interestingly, a direct relationship between lower eLogD and higher KS (at pH 7.4) was not observed for compounds **2** and **8**, which have a lower eLogD than **1**, but presented lower KS at pH 7. Compound **4** is the most water‐soluble, exhibiting a KS > 130 μM at both pH 2.0 and 7.4. Additionally, Compound **5** also achieved a KS > 130 μM at pH 2.0, and compounds **1** and **9** demonstrated a KS > 80 μM at both pH values. Overall, the compounds showed higher solubility at pH 2.0 compared to pH 7.4. This finding can be attributed to the increased ionization capacity of the basic sites within these molecules (such as the pyridine fragment) under more acidic pH conditions.

Metabolic stability assays in mouse liver microsomes (MLM) and human liver microsomes (HLM) revealed insights into the structure–metabolism relationship. Overall, the MLM and HLM data were differentially impacted by the chemical modifications, which was not necessarily unexpected given the distinct species involved. Consequently, we prioritized the discussion of the primary findings, placing greater emphasis on the MLM data, as our objective was to identify compounds with suitable ADME profiles for a subsequent in vivo mouse study.

The initial hit **1**, showed moderate metabolic stability, with a half‐life slightly over 1 h and clearance values near 40 µL/min/mg for both MLM and HLM. In an attempt to improve metabolic stability, some changes were designed in toluyl fragment of hit **1.** Compound **2**, a phenyl derivative lacking the methyl group of the toluyl fragment in hit **1**, demonstrated improved metabolic stability in MLM, with approximately a twofold decrease in clearance and a twofold increase in half‐life, suggesting that the toluyl fragment is more susceptible to metabolism than the phenyl fragment. However, in HLM, compound **2** exhibited reduced stability, with significantly increased clearance (CL_int_ = 218.40 µL/min/mg) and a reduced half‐life (*t*_1/2_ = 12.7 min).

Compounds **3–5** were designed as monosubstituted *N*‐arylpiperazine derivatives to assess the influence of electronic effects. These derivatives replaced the *ortho*‐methyl group in hit **1** with electron‐withdrawing groups (F, Cl, and CF_3_). Metabolic stability in MLM remained comparable to hit **1**, except for compound **3** (*ortho*‐CF_3_), which showed increased clearance (CL_int_ = 100.80 µL/min/mg) and reduced half‐life (*t*_1/2_ = 27.51 min). In HLM, compound **3** exhibited a twofold improvement in metabolic stability compared to hit **1** (CL_int_ = 26.80 µL/min/mg, *t*_1/2_ = 103.45 min). The substitution of the methyl group (initial hit **1**) with a trifluoromethyl group (**3**) can potentially improve metabolic stability by eliminating a site susceptible to oxidative metabolism (the benzylic carbon in **1**). However, this chemical modification also increases lipophilicity (eLogD = 4.07 versus 4.34 for compounds **1** and **3**, respectively). Consequently, this increase in lipophilicity may be accompanied by a reduction in metabolic stability.

Compound **5** (*ortho*‐Cl) was not only the most potent derivative in the series against chloroquine‐sensitive strain (IC_50_^*Pf*3D7^ = 0.4 μM), but also the most cytotoxic against HepG2 cells. Its metabolic stability showed minimal variation compared to hit **1**, with a slight decrease in MLM and a modest improvement in HLM. Replacing the *ortho*‐methyl group in hit **1** with a *para*‐methoxy group (compound **6**) had minimal impact on metabolic stability in MLM but resulted in a significant increase in clearance and a reduced half‐life in HLM. Insertion of an endocyclic nitrogen into the phenyl ring proved beneficial for metabolic stability. The isomers **7** and **8**, which feature an endocyclic nitrogen and *ortho*‐ or *para*‐CF_3 _ substituents, demonstrated enhanced stability in MLM when compared to compound **3** (which has no endocyclic nitrogen). This improved stability can likely be attributed to the lower lipophilicity of **7** and **8** (eLogD < 4) and reduction of the electron density in the aromatic ring. Compound **8** (*para*‐CF_3_) exhibited even more improvement, with a clearance value twofold less (CL_int_ = 42.40 µL/min/mg) and more than double the half‐life (*t*_1/2_ = 65.39 min) compared to compound **3**, suggesting that the CF_3_ position significantly influences MLM stability, with the *para* position being more favorable to metabolic stability. However, in HLM, these changes have little influence in metabolic stability wherein clearance and half‐life of compounds **7** and **8** were comparable to those of compound **3**.

The incorporation of endocyclic nitrogen atoms into the aromatic ring demonstrated a significant impact on metabolic stability, greatly improving the understanding of the structure–metabolism relationship. While introducing a single nitrogen atom (compounds **7** and **8**) already showed substantial benefits, the subsequent addition of a second nitrogen atom (compounds **9** and **10**) further enhanced the metabolic profile. Among the evaluated compounds, the pyrazinyl derivative **9** stood out with the most favorable metabolic stability profile, also presenting the lowest lipophilicity in the set (eLogD = 2.35). In MLM, **9** exhibited a remarkably low intrinsic clearance (Cl_int_  < 12μL/min/mg) and an extended half‐life of 10.5 h. These values represent a more than threefold reduction in clearance and nearly a tenfold increase in half‐life compared to the initial hit **1**, making **9** a highly promising candidate for in vivo evaluation. Furthermore, **9** retained its superior stability in HLM, showing clearance approximately half that of hit **1** (CL_int_  = 25.20 μL/min/mg) and a twofold increase in half‐life (*t*_1/2_  = 110.02 min).

To investigate the effect of endocyclic nitrogen position, the pyrimidyl derivative (**10**) was designed. This compound showed a comparable lipophilicity value (eLogD = 2.58) and retained the favorable metabolic stability observed with **9**, particularly in HLM, where clearance and half‐life values remained comparable. Specifically, **10** displayed an intrinsic clearance of 13.60 μL/min/mg in MLM, slightly higher than **9** but still indicative of excellent metabolic stability. Its half‐life, though shorter than that of **9**, remained within an acceptable range, confirming its status as a metabolically stable candidate.

The enhanced stability for **9** and **10** in both MLM and HLM can be attributed to two combined effects. Primarily, the electronic effects exerted by the more electronegative nitrogen atoms induce an electron‐withdrawing effect that reduces the electron density of adjacent carbons. This reduction can make those carbons less susceptible to enzymatic attacks and oxidative metabolic reactions. Crucially, the significant decrease in lipophilicity (eLogD < 2.6) observed in compounds containing two endocyclic nitrogen atoms also contributes to their heightened metabolic resistance [19, 20]. Collectively, these factors create a molecular environment less favorable for metabolic degradation, thereby validating this structural strategy. Compounds **9** and **10** displayed the best drug‐like profile in the series, combining optimal values for lipophilicity, permeability, aqueous solubility, and metabolic stability. However, **9** is the more promising lead candidate as it is both more active and less cytotoxic than **10**. Additionally, **7** also stands out as a potential lead candidate due to its appropriate ADME values and for being one of the most active and selective compounds against *P. falciparum*. These results underscore the optimization of this chemical class against malaria, achieved through chemical modifications to the toluyl fragment of the initial hit **1**. These changes led to the maintenance of potent biological activity against *P. falciparum* while enhancing the ADME properties, resulting in **7** and **9**, which are strong candidates to exhibit improved bioavailability and efficacy in in vivo malaria models.

### 2.3 Synthesis of 7 and 9

**SCHEME 1:**
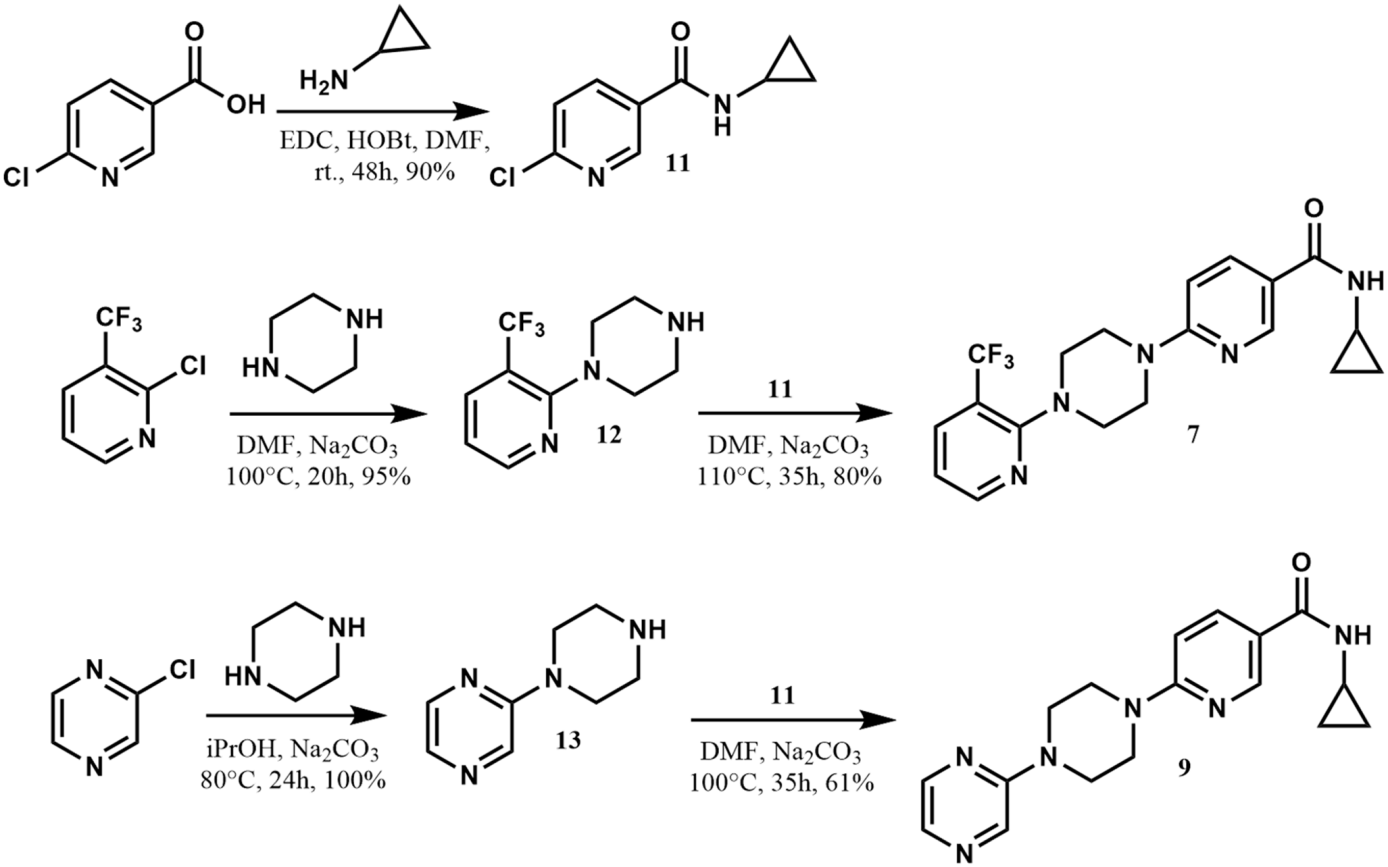
Scale‐up synthesis route for compounds **7** and **9**.

In the present work, a three‐step convergent synthetic route was employed for the preparation of **7**, achieving an overall yield of 68%. This represents a substantial improvement over the previously reported three‐step linear sequence, which afforded the product in only an 11% overall yield. In this optimized approach, intermediates **11** and **12** were synthesized separately and subsequently linked via a nucleophilic aromatic substitution (SNAr) reaction to furnish **7** in significantly improved yields. Intermediate **11** was first obtained via amide coupling between *p*‐chloronicotinic acid and cyclopropanamine, in the presence of EDC and HOBt at room temperature, yielding 90%. Intermediate **12** was then prepared through an SNAr reaction between 2‐chloro‐3‐(trifluoromethyl)pyridine and piperazine under basic conditions at 100°C for 20 h, affording 95% yield. The final SNAr reaction between intermediates **11** and **12**, also performed under basic conditions at 100°C provided the target compound **7** in 80% yield. This convergent route proved to be substantially more efficient than the previously reported method and was thus successfully utilized for the necessary larger‐scale production. For compound **9**, we utilized the previously established three‐step linear synthetic route. The synthesis commenced with the preparation of intermediate **13**. This intermediate was efficiently obtained via an SNAr reaction between 2‐chloropyrazine and piperazine in isopropanol, using sodium carbonate as the base over a 24 h period, furnishing intermediate **13** quantitatively. The final compound **9** was subsequently synthesized through an SNAr reaction between intermediates **11** and **13** under basic conditions at 100°C, affording the target compound in a 61% yield (Scheme 1). Significantly, the optimized larger‐scale route proved to be more efficient, leading to a final overall yield of 55%, which is a substantial improvement compared to the 28% overall yield achieved in the smaller‐scale preparation.

### 2.4 In Vivo Efficacy Studies Against Malaria

**FIGURE 1:**
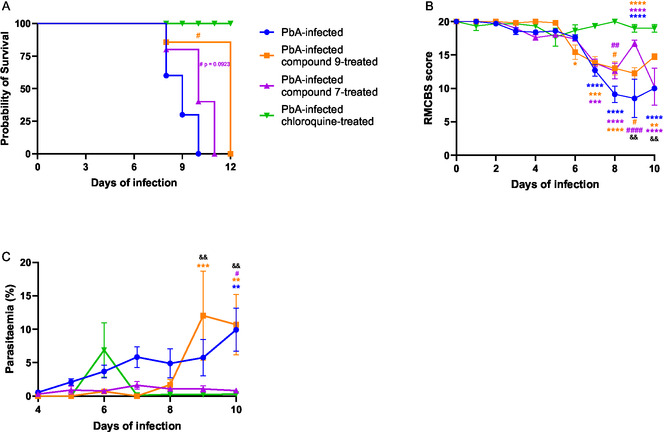
Survival rate, RMCBS and parasitemia of C57BL/6 mice treated with 100mg/kg/day of **7** (triangle symbols) or **9** (squares symbols) diluted in vehicle (PBS 0.16% Tween 80), 10% DMSO, 40% PEG400 (Polyethylene Glycol 400, Sigma‐Aldrich) 1 day after inoculation of animals with 1 × 10^5^ red blood cells infected with PbA and continued until the 8th day of infection, totaling 7 days of treatment (*n* = 5 per group). A group of mice was treated similarly with Chloroquine 10mg/kg/day as a reference drug.. Control mice were infected and treated with vehicle only (*n* = 5 per group). The survival (A), RMCBS score (B), and parasitemia (C) were evaluated. Data are presented as mean of each group ± SEM. The survival rate was analyzed by Chi‐square test; RMCBS score and parasitemia by two‐way ANOVA, followed by Tukey's multiple comparisons test. (*) Statistical differences in relation to the Chloroquine‐treated group are pointed according to the color of the symbol of each group, being orange compared to group **9** and purple compared to group **7**. *p < 0.05, **p < 0.01, ***p < 0.001, ****p < 0.0001. (#) Statistical differences in relation to the PbA‐infected group are pointed according to the color of the symbol of each group, being orange compared to group **9** and purple compared to group **7**.^.^^#^ *p* < 0.05, ^##^ *p* < 0.01, ^###^ *p* < 0.001, ^####^ *p* < 0.0001. Statistical differences between **7** and **9**‐treated infected groups^.^^&&^ *p* < 0.01.

Compounds **7** and **9** were selected as the most promising lead candidates because they demonstrated the best balance between the in vitro pharmacodynamic and pharmacokinetic properties evaluated. Both compounds were assessed in vivo in C57BL/6 mice infected with *P. berghei* ANKA, a lethal strain associated with cerebral malaria, to investigate their antimalarial effects. Daily doses of 100 mg/kg of each compound were administered intraperitoneally to the mice from day one to day eight of infection, totalizing 7 days of treatment. As controls, one group of infected mice was treated with chloroquine (10 mg/kg/day), and another group received the vehicle only. Parasitemia, clinical signs of cerebral malaria, and survival rate were evaluated (Figure 1).

Animals treated with **7** and **9** showed a higher survival rate compared to infected nontreated animals, exhibiting approximately 80% survival on the last day of administration (day 8) compared to 60% survival of PbA‐infected group in the same time point (Figure 1A). In order to assess specific clinical signs of cerebral malaria, animals were evaluated using the rapid murine coma and behavior scale (RMCBS) starting from day 4 postinfection (p.i.; Figure 1B). The RMCBS is a quantitative scale that evaluates 10 parameters (gait, balance, motor performance, body position, limb strength, touch escape, pinna reflex, toe pinch, aggression, and grooming) and is scored from 0 to 20, with a 0 score correlating with the lowest function and a 20 score with the highest, 20 being the maximal score [21]. The animals treated with **7** and **9** also showed a better RMCBS score than the nontreated animals, indicating that the treated animals did not develop cerebral malaria.

Parasitemia in the infected mice was assessed daily from day 4 until day 10 p.i. (Figure 1C). A significant reduction in parasitic load was observed in the group treated with compound **7**, achieving reductions of 78.0%, 81.4%, and 91.7% on days 8, 9, and 10 of infection, respectively, when compared to the infected nontreated control group. Conversely, animals treated with compound **9** exhibited a notable reduction in parasitemia on day 7 of infection, followed by a moderate reduction of 65.2% on day 8 of infection. However, this effect was not sustained after the end of treatment, as parasitemia increased from day 9 onward. In contrast, the group treated with compound **7** continued to reduce parasitemia. These results highlight compound **7** as a lead compound against malaria, demonstrating over 90% reduction in parasitemia in infected mice.

## 3 Conclusion

This study achieved the hit‐to‐lead optimization of the pyridylpiperazine class, yielding derivatives with significantly enhanced ADME profiles while maintaining potent antiplasmodial activity against *P. falciparum*. The systematic evaluation of structure–ADME relationships demonstrated that chemical modifications to the toluyl fragment of the initial hit 1, particularly the introduction of endocyclic nitrogen atoms, were highly effective. The strategic incorporation of two endocyclic nitrogen atoms led to the most significant improvement in metabolic stability, primarily through favorable electronic effects and a substantial reduction in lipophilicity (eLogD < 2.6). This process identified compounds **7** and **9** as the most promising lead candidates, exhibiting an optimal balance of permeability, aqueous solubility, and exceptional metabolic stability in liver microsomes. Notably, the pyrazinyl derivative **9** achieved a nearly tenfold increase in half‐life in MLM compared to the initial hit **1**, demonstrating a highly favorable pharmacokinetic outlook. Most importantly, the therapeutic potential of these optimized compounds was validated in a stringent in vivo model of cerebral malaria (*P. berghei* ANKA). Both compounds significantly protected mice from clinical signs of cerebral malaria (indicated by improved RMCBS scores) and increased the survival rate to approximately 80% by the last day of treatment. Compound **7** emerged as the most efficacious in vivo agent, sustaining parasitaemia reduction even after the cessation of treatment and achieving a remarkable 91.7% reduction in parasitic load by day 10. In summary, these findings highlight **7** and **9** as viable, potent, and metabolically stable lead candidates for further preclinical development against drug‐resistant malaria. Future efforts should focus on exploring chemical modifications on other fragments of the molecular scaffold to further refine the overall safety and efficacy profiles, along with elucidating the precise mechanism of action of this promising pyridylpiperazine class.

## 4 Materials and Methods

### 4.1 In Vitro ADME Experiments

All ADME analyses were performed using liquid chromatography‐tandem mass spectrometry (LC‐MS/MS). The LC‐MS/MS system used consisted of a Prominence UFLC (Shimadzu Corporation, Kyoto, Japan) chromatography system, interfaced with a LCMS‐8045 triple quadrupole mass spectrometer (Shimadzu Corporation, Kyoto, Japan) equipped with an electrospray ionization source (ESI).

### 4.2 *Experimental Lipophilicity Determination via Distribution Coefficient* (*eLogD*_7.4_)

For the determination of the distribution coefficient (eLogD), a methodology based on the retention time of molecules in the stationary phase was used. The chromatogram was achieved on a Supelco Ascentis express RP amide HPLC column (5 cm × 2.1 mm × 2.7 µm), using 5% methanol in 10 mM ammonium acetate (pH 7.4 (A)) and 100% methanol (B) as mobile phases. The mobile phase was eluted in binary gradient mode, as follows: 0 min: 95% A; 0.3 min: 100% A; 5.2 min: 0% A; 5.6 min: 0% A; 5.8 min: 100% A; 7.0 min: 100% A. The total run time was 7 min, and 5 µL of each sample was injected. The solutions of tested compounds were prepared at a concentration of 1.0 mg/mL in compound prepared by adding the stock solution to a (1:1) mix of mobile phases A:B (internal standard at 200 nM), and the DMSO concentration was lower than 2%. The lipophilicity of compounds was assessed by injecting individually the test compounds and a series of eight commercial drugs, covering a LogD range of −1.86 to 6.1 (acyclovir: −1.86, atenolol: 0.16, antipyrine: 0.38, fluconazole: 0.50, metoprolol: 1.88, ketoconazole: 3.83, tolnaftate: 5.40, and amiodarone: 6.10) [22, 23, 24]. The retention time (in minutes) of each of the eight standards was plotted against their LogD values, and the resulting equation for the calibration curve (*y* = *mx* + *b*) was used to calculate the eLogD values for the compounds from their retention time.

### 4.3 Kinetic Solubility

To determine kinetic solubility, stock solutions of test compounds and controls (10 mM in DMSO) were transferred to two 96‐well plates (incubation plates) in duplicates. PBS pH 7.4 or 2.0 (final concentration of 250 µM) was added to each well of the plates, and the DMSO concentration was kept lower than 2.5%. The plates were sealed and shaken for 24 h (200 rpm/25°C). The precipitates on the incubation plate were removed by centrifugation (15 min/3000 rpm/25°C), and the supernatant fractions were quantified by LC‐MS/MS. An intermediate standard solution diluted at 0.5 mM in acetonitrile:water (1:1) was prepared from a 10 mM standard solution. A calibration curve was prepared for each of the test compounds and controls by diluting several times the intermediate standard solution to reach the desired concentrations of 50, 40, 20, 2, and 1 µM. The resulting equation for the calibration curve (y = mx + b) was used to calculate the experimental concentration values for the compounds. The chromatogram for analysis was achieved on a Supelco Ascentis express C18 column (3 cm × 2.1 mm × 5 µm), using water + 0.05% formic acid (A) and acetonitrile + 0.05% formic acid (B) as mobile phases. The mobile phase was eluted in binary gradient mode, and the gradient was as follows: 0 min: 98% A; 1.2 min: 2% A; 2.0 min: 2% A; re‐equilibration time: 0.6 min, 98% A. The total run time was 2 min, and 5 µL of each sample were injected and eluted at a flow rate of 0.6 mL/min. The drugs amiodarone, diclofenac, chloramphenicol, and itraconazole were used as positive controls.

### 4.4 *Parallel Artificial Membrane Permeability Assay* (*PAMPA*)

The PAMPA assay was carried out in a 96‐well precoated PAMPA plate system (Corning Gentest # 353015). Solutions of the compounds were prepared by diluting the stock solutions (10 mM) in phosphate buffered saline (PBS) at pH 6.5 to a final concentration of 10 µM, then added to the donor portion of the plate (300 µL/well), while PBS at pH 7.4 (200 µL/well) was added in the acceptor portion. The DMSO concentration in the solutions was lower than 1%. The two portions of the plate were then assembled, and the system was incubated (5 h/37°C). Samples of the initial donor solution (T0) were collected and stored at −20°C. At the end of the incubation, samples were collected from the donor and acceptor plates, then added to plates containing a quenching solution (10% water and 90% methanol: acetonitrile (50:50) + 50 nM tolbutamide), and the T0 samples were treated similarly. The final concentrations of compounds in the donor, acceptor and T0 wells were quantified by LC‐MS/MS. The chromatograms for analysis were achieved on a Supelco Ascentis express C18 column (3 cm × 2.1 mm × 5 µm). The mobile phases consisted of water + 0.1% formic acid (A) and acetonitrile + 0.1% formic acid (B). The mobile phase was eluted in binary gradient mode, and the gradient was as follows: 0 min: 95% A; 0.05 min: 95% A; 0.3 min: 2% A; 0.7 min: 2% A; 0.8 min: 95% A; 1.15 min: 95% A; 2.0 min: 95% A. The total run time was 2 min, and 10 µL of each sample were injected and eluted at a flow rate of 0.7 mL/min. The results were used to calculate an effective permeability (*P*_*e*_) value. All PAMPA assays were performed in triplicates. The drugs nadolol and metoprolol were used as positive controls.

### 4.5 *Metabolic Stability in Mouse* (*MLM*) *and* *HLM*

Metabolic stability was evaluated in pooled HLM (20 mg/mL, GIBCO) and pooled CD1 MLMs (20 mg/mL, GIBCO). Solutions of the compounds were prepared at a concentration of 0.5 µM and incubated with 0.25 mg/mL liver microsomes in PBS of pH 7.4. The DMSO concentration in the solutions was lower than 1%. The reactions were started by adding 0.5 µM NADPH cofactor. Samples were taken at timepoints 0 (immediately following the addition of the cofactor), 5, 10, 20, 30, and 60 min. The reactions were stopped by adding a quenching solution containing acetonitrile:methanol (1:1) (internal standard at 50 nM). The samples were then centrifuged (3500 rpm/30 min) to obtain pellets of precipitated microsomal protein. The supernatant fractions were quantified by LC‐MS/MS. Peak area ratios (analyte/internal standard) were converted to % remaining using the area ratio at time 0 as 100%. Half‐life (*t*_1/2_ = ln(2)/*k*) in minutes and intrinsic clearance (Cl_int_ = *k* × 1000/(0.25)) in µL/min/mg were calculated using a nonlinear regression of % remaining versus incubation time. From this plot, the slope (*k*) was determined. The chromatograms for analysis were achieved on a Supelco Ascentis express C18 column (3 cm × 2.1 mm × 5 µm). The mobile phases consisted of water +0.1% formic acid (A) and acetonitrile + 0.1% formic acid (B). The mobile phase was eluted in binary gradient mode, and the gradient was as follows: 0 min: 95% A; 0.05 min: 95% A; 0.3 min: 2% A; 0.7 min: 2% A; 0.8 min: 95% A; 1.15 min: 95% A; 2.0 min: 95% A. The total run time was 2 min, and 10 µL of each sample were injected and eluted at a flow rate of 0.7 mL/min. Microsomal stability tests were performed in triplicates. The drugs imipramine, verapamil, and warfarin were used as positive controls.

### 4.6 In Vivo Efficacy Experiments

#### 4.6.1 Animals

8–12‐week‐old female C57BL/6 mice were used in this investigation. The mice were bred and maintained in specific pathogen‐free conditions at the animal facility (Rede de Biotérios de Roedores ‐ REBIR/UFU) at the Universidade Federal de Uberlândia, with 12 h light/dark cycles and free access to food and filtered water. All animal experiments were performed in accordance with the Brazilian Government's ethical standards and were approved by the Comitê de Ética na Utilização de Animais (CEUA) of the Universidade Federal de Uberlândia, under protocol no. 018/21.

#### 4.6.2 Parasite Strain

The PbA strain expressing green fluorescent protein (GFP) was used to infect the animals in this study. Parasite strain was maintained in liquid nitrogen and defrosted to perform passages in C57BL/6 mice. Vials containing known amounts of infected RBCs resuspended in PBS were prepared and stored at −80°C until the time of infection.

#### 4.6.3 Experimental Procedure

Mice were intraperitoneally inoculated with 1 × 10^5^ RBCs infected with PbA. 1 day after infection, groups of five mice were inoculated with compound **7** or **9**, 100 mg/kg/day diluted in vehicle [PBS 0.16% Tween 80, 10% DMSO, 40% Polyethylene Glycol 400 (PEG400, Sigma‐Aldrich)], and treatment persisted for 7 days. Chloroquine was used as reference drug at dose of 10 mg/kg/day, with the animals treated similarly to compounds **7** and **9**, starting 1 day after infection and lasting 7 days. Control mice were treated i.p. with the vehicle only.

Survival and clinical signs were evaluated daily. In order to assess specific clinical signs of cerebral malaria, animals were evaluated according to RMCBS as previously described by Carroll et al. starting from day 4 p.i. [21].

For parasitaemia determination a drop of tail blood was collected in 500 μL of PBS and samples were fixed with formaldehyde 4% in PBS and stored at 4°C until processing. Parasitaemia of infected RBCs (iRBCs) was assessed daily starting from day 4 until day 10 p.i., detecting iRBCs GFP^+^ by flow cytometry using the Accuri C6 flow cytometer (BD Biosciences, San Jose, CA, USA) and data were evaluated using FlowJo V10 software. A total of 30,000 gated events were acquired and recorded for analysis from each sample. In addition, the parasitaemia was also evaluated in thin blood smears (Instant Prov Rapid haematological staining ‐ NewProv, Brazil) by light microscopy.

#### 4.6.4 Statistical Analysis

The Kaplan–Meier method was used to compare the survival rates of the experimental groups and the percent survival was compared using Chi‐square test. RMCBS score and parasitaemia were analyzed by two‐way ANOVA followed by Tukey's multiple comparisons test. Values of *p* < 0.05 were considered statistically significant.

### 4.7 Synthesis

#### 4.7.1 General Information

Unless specified, all reactions were performed using commercial reagents and solvents were used without further purification. Flash column chromatography was performed using either Aldrich silica gel (35–70 mesh). Analytical thin‐layer chromatography was performed on chromatography aluminum sheets impregnated with silica‐gel 60 F254 (Sigma‐Aldrich) and plate revelation was achieved using UV light (254 nm) and/or iodine atmosphere. ^1^H, ^13^C proton‐decoupled and ^13^C APT NMR spectra were acquired in CDCl_3_ at 400 MHz (^1^H) and 100 MHz (^13^C and ^13^C APT) (Bruker Ascend 400). Chemical shifts (δ) are reported in ppm using residual solvent peak as an internal standard (CDCl_3_: 7.26 ppm, TMS: 0.00 ppm for ^1^H NMR spectra, and CDCl_3_: 77.16 ppm for ^13^C and ^13^C APT NMR spectra). Peak multiplicity was reported using the following abbreviations: s = singlet, d = doublet, dd = doublet of doublets, m = multiplet. The multiplicity is followed by the coupling constant(s) in Hz and integration. Exchangeable protons were not observable on some NMR spectra. For ^13^C APT NMR spectra, peaks pointing upwards correspond to primary and tertiary carbons, and peaks pointing down wards correspond to secondary and quaternary carbons. High resolution mass spectrometry (HRMS) was performed on a Q‐TOF geometry Impact II (Bruker Daltonics Corporation, Germany) equipped with an ESI. The Q‐TOF analyzer was calibrated using a sodium formate solution (10 mmol L‐1; isopropanol:water; 1:1; v:v) and operated with the following parameters: the capillary voltage was operated in positive ion mode, set at 4500 V with an end plate offset potential of 500 V, nebulizer pressure, 0.4 bar; dry gas flow, 4 L/min, dry temperature, 180°C; acquisition spectra rate was 1.00 Hz, monitoring a mass range from 50 to 700 m/z. The samples were resuspended in 1 mL of MeOH LC‐MS grade, shaken in vortex equipment for 30 s, then a 5 L aliquot was solubilized in 500 μ L of MeOH LC‐MS and injected directly into the mass spectrometer using a syringe pump (KDS Legato 100, KD Scientific, Holliston, MA, USA) at a flow rate of 20 μL/min. Compound purity was verified by high‐performance liquid chromatography (HPLC) using a Shimadzu SPD‐M10A vp instrument. Compounds **7** and **9** are >97% pure by HPLC analysis. The NMR, HRMS spectra and HPLC chromatogram are available in the supporting information.

#### 4.7.2 Synthesis of Intermediates 11–13

##### 4.7.2.1 6‐Chloro‐N‐cyclopropylnicotinamide (11)

*p*‐Chloronicotinic acid (1.0 equiv., 2.0 g, 12.69 mmol.), EDC (1.2 equiv., 2.91 g, 15.23 mmol), and HOBt (0.1 equiv., 0.1714 g; 1.269 mol) were solubilized in DMF (12 mL). Cyclopropanamine (1.2 equiv., 0.8697 g, 15.23 mol) was added, and the solution was stirred at room temperature until completion of the reaction. The reaction was treated with cold water, and the aqueous layer was extracted with EtOAc (4 × 20 mL). The combined organic layers were washed with brine, dried over Na_2_SO_4_, filtered, and the solvent was evaporated under reduced pressure. The residue was then purified by flash column chromatography using DCM/MeOH (98:2) as eluent.

(**11**): White solid, 2.24 g, 90 %. **^1^** **H NMR (400** **MHz, CDCl** **_3_** **)**: δ 8.69 (dd, *J* = 2.5, 0.5 Hz, 1H), 8.06 (dd, *J* = 8.3, 2.5 Hz, 1H), 7.38 (dd, *J* = 8.3, 0.5 Hz, 1H), 6.56 (s, 1H), 3.01–2.77 (m, 1H), 0.99–0.78 (m, 2H), 0.75–0.56 (m, 2H). ^**1** **3**^ **C NMR (101** **MHz, CDCl** **_3_** **)**: δ 166.10, 154.37, 147.97, 138.08, 129.13, 124.49, 23.44, 6.89.

##### 4.7.2.2 1‐(3‐(Trifluoromethyl)pyridin‐2‐yl)piperazine (12)

2‐Chloro‐3‐(trifluoromethyl)pyridine (1.0 equiv., 500 mg, 2.754 mmol) was solubilized in DMF (3 mL). Piperazine (3.0 equiv., 712 mg, 8.2625 mmol) and Na_2_CO_3_ (3.0 equiv., 875 mg, 8.2625 mmol) were added, and the reaction was stirred for 20 h at 100°C. Then, it was treated with cold water (15 times the amount of solvent). The aqueous layer was extracted with EtOAc (3 × 15 mL), and the combined organic layers were washed with brine, dried over Na_2_SO_4_, filtered, and the solvent was evaporated under reduced pressure to give 12 without further purification needed.

**12:** Colorless oil, 605 mg, 95%. ^**1**^ **H NMR (400 MHz, CDCl**_**3**_ **):** δ 8.44 (d, *J* = 3.6 Hz, 1H), 7.86 (dd, *J* = 7.4, 1.5 Hz, 1H), 6.98 (dd, *J* = 7.4, 4.8 Hz, 1H), 3.31–3.23 (m, 4H), 3.05–2.98 (m, 4H). ^**13**^ **C APT NMR (101 MHz, CDCl**_**3**_ **):** δ 160.1, 151.1, 137.3 (q, *J* = 5.1 Hz), 124.1 (q, *J* = 272.6 Hz), 116.9, 52.2, 46.2.

##### 4.7.2.3 2‐(Pirazin‐1‐yl)‐piperazine (13)

2‐Chloropyrazine (300 mg, 2.619 mmol) was solubilized in iPrOH (0.5 M). Piperazine (676.8 mg, 7.857 mmol, 3 eq) and Na_2_CO_3_ (832.7 mg, 7.857 mmol, 3 eq) were added, and the reaction was stirred for 24 h at 80°C. iPrOH was evaporated under reduced pressure, and the crude residue was purified by flash column chromatography (DCM/MeOH (9:1) to (8:2) gradient).

**13:** Yellow oil, 435.7 mg, 100%. ^**1**^ **H NMR (400 MHz, CDCl**_**3**_ **):** δ 8.11 (d, *J* = 1.2 Hz, 1H), 8.04 (dd, *J* = 2.7, 1.4 Hz, 1H), 7.82 (d, *J* = 1.6 Hz, 1H), 3.59–3.49 (m, 4H), 3.03–2.91 (m, 4H), 1.93 (s, 1H). ^**13**^ **C APT NMR (101 MHz, CDCl_3_):** δ 155.3, 141.8, 132.9, 131.1, 45.8, 45.6.

#### 4.7.3 Synthesis of Derivatives 7 and 9

##### 4.7.3.1 N‐Cyclopropyl‐6‐(4‐(3‐(trifluoromethyl)pyridin‐2‐yl)piperazin‐1‐yl)nicotinamide (7)

1‐(3‐(Trifluoromethyl)pyridin‐2‐yl)piperazine **12** (1.2 equiv., 354 mg, 1.53 mmol) was dissolved in DMF (3.0 mL), and 6‐chloro‐*N*‐cyclopropylnicotinamide **11** (1.0 equiv., 250 mg, 1.28 mmol) and Na_2_CO_3_ (2.0 equiv., 270 mg, 2.55 mmol) were added. The reaction mixture was stirred at 110°C for 35 h. After completion, the mixture was cooled to room temperature and treated with cold distilled water (approximately 10 times the reaction volume), resulting in the formation of a solid precipitate. The solid was filtered, washed with distilled water, and purified by recrystallization from hexane under heating to afford the desired product.

**(7):** Yellow solid, 400 mg, 80%. ^**1**^ **H NMR (400 MHz, CDCl**_**3**_ **):** δ 8.52 (s, 1H), 8.45 (d, *J* = 4.6 Hz, 1H), 7.91–7.88 (m, 2H), 7.08–6.99 (m, 1H), 6.65 (d, *J* = 9.0 Hz, 1H), 6.06 (s, 1H), 3.84–3.74 (m, 4H), 3.44–3.36 (m, 4H), 2.89–2.86 (m, 1H), 0.88–0.83 (m, 2H), 0.62–0.60 (m, 2H). ^**13**^C APT NMR (101 MHz, CDCl_**3**_) δ 160.42, 159.62, 151.11, 151.06, 147.15, 137.29, 137.24, 136.78, 125.27, 118.93, 117.38, 105.74, 50.34, 44.86, 22.99, 6.85. **HRMS (ESI+):** calcd. for C_19_H_21_F_3_N_5_O^+^ [M + H]^+^ = 392.1693 *m/z*, found [M + H]^+^ = 392.1683 *m/z*, error = 2.55 ppm. HPLC purity: 97.32%.

##### 4.7.3.2 N‐Cyclopropyl‐6‐(4‐(pyrazin‐2‐yl)piperazin‐1‐yl)nicotinamide (9)

2‐(Piperazin‐1‐yl)‐piperazine (13) (1.0 equiv., 150 mg, 0.9134 mmol) was dissolved in DMF (2.0 mL), and 6‐chloro‐*N*‐cyclopropylnicotinamide **11** (1.2 equiv., 215.5 mg, 1.096 mmol) and Na_2_CO_3_ (2.0 equiv., 193.64 mg, 1.826 mmol) were added. The reaction mixture was stirred at 100°C for 35 h. After completion, the mixture was cooled to room temperature and treated with cold distilled water (approximately 10 times the reaction volume), resulting in the formation of a solid precipitate. The solid was filtered, washed with distilled water, and dry under reduced pressure to give **9** without further purification needed.

**9:** White solid, 182 mg, 61%. ^**1**^ **H NMR (400 MHz, CDCl**_**3**_ **):** δ 8.53 (d, *J* = 2.3 Hz, 1H), 8.17 (s, 1H), 8.09 (s, 1H), 7.92 (dd, *J* = 9.0, 2.3 Hz, 1H), 7.89 (d, *J* = 2.3 Hz, 1H), 6.64 (d, *J* = 9.0 Hz, 1H), 6.09 (s, 1H), 3.82–3.80 (m, 4H), 3.75–3.73 (m, 4H), 2.94–2.83 (m, 1H), 0.88–0.83 (m, 2H), 0.69–0.54 (m, 2H). ^**13**^ **C APT NMR (101 MHz, CDCl**_**3**_ **):** δ 160.17, 154.90, 147.36, 141.94, 137.06, 133.55, 131.12, 119.43, 105.80, 44.31, 44.12, 23.18, 7.01. **HRMS (ESI+):** calcd. for C_17_H_21_N_6_O^+^ [M + H]^+^ = 325.1771 *m/z*, found [M + H]^+^ = 325.1769 *m/z*, error = 0.62 ppm. **HPLC purity:** 99.52%.

## Funding

This study was supported by Fundação de Amparo à Pesquisa do Estado de Minas Gerais (APQ‐01116‐21, APQ‐01705‐21, RED00110‐23), Conselho Nacional de Desenvolvimento Científico e Tecnológico (300079/2025‐7), and Fundação de Amparo à Pesquisa do Estado de São Paulo (13/07600‐3).

## Conflicts of Interest

The authors declare no conflicts of interest.

## Supporting information

NMR and HRMS spectra of compounds **7** and **9** can be found in the Supporting Information.

## Data Availability

Data sharing not applicable to this article as no datasets were generated or analyzed during the current study.
