# Supplementary material for: Hit‐To‐Lead Optimization of a Pyridylpiperazine Class Against Malaria: Pharmacokinetic Profile and In Vivo Efficacy of Optimized Compounds
Source: ChemMedChem. 2026 Jul 8;21(13):e70373. doi: 10.1002/cmdc.70373 (PMC13346333; doi:10.1002/cmdc.70373)
Supplement: Supplementary file 1 — NMR and HRMS spectra of compounds 7 and 9 can be found in the Supporting Information. [file CMDC-21-e70373-s001.pdf]

Supporting Information

Hit-to-lead optimization of a pyridylpiperazine class against malaria: Pharmacokinetic profile and *in vivo* efficacy of optimized compounds

Douglas Davison da Silva Oliveira[a], Duarte Eduardo Pereira[b], Rafael Consolin Chelucci[c], Simone Michelan[c], Júlia Maria Fernandes Pituba[b], Adriano D. Andricopulo[c], Leonardo L. G. Ferreira[c], Neide Maria Silva[b], Celso de Oliveira Rezende Júnior[a]*

* Corresponding authors: celso@ufu.br

1. **NMR of ^{1}H and ^{13}C of compounds 7, 9 and 11-13 and HRMS of compounds 7 and 9**

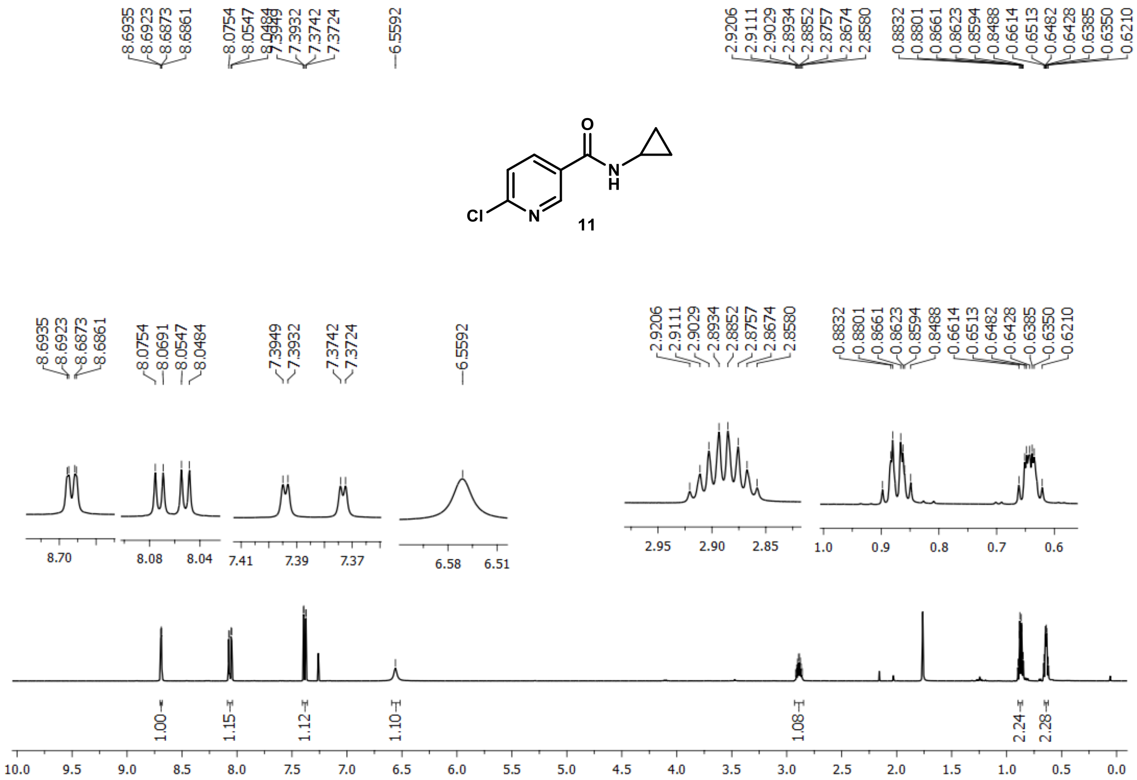

Figure S1 – ^{1}H NMR spectrum of **11** (400 MHz, $CDCl_3$)

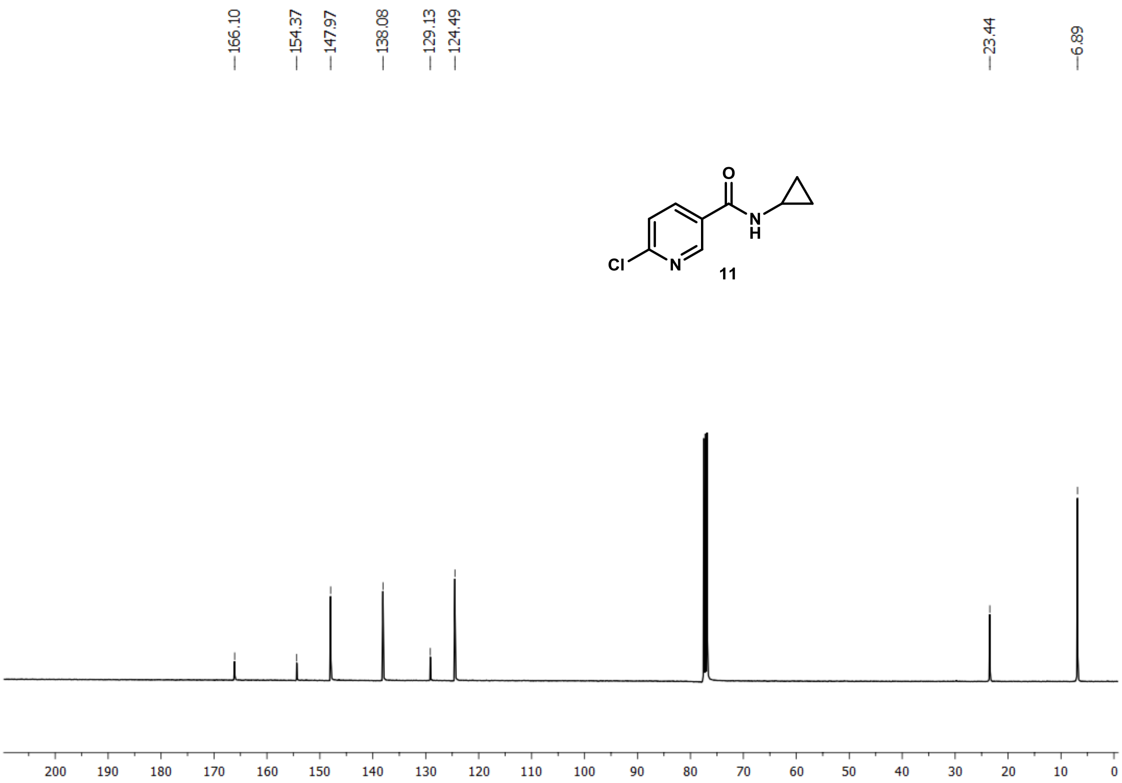

Figure S2 – ^{13}C NMR spectrum of **11** (101 MHz, $CDCl_3$)

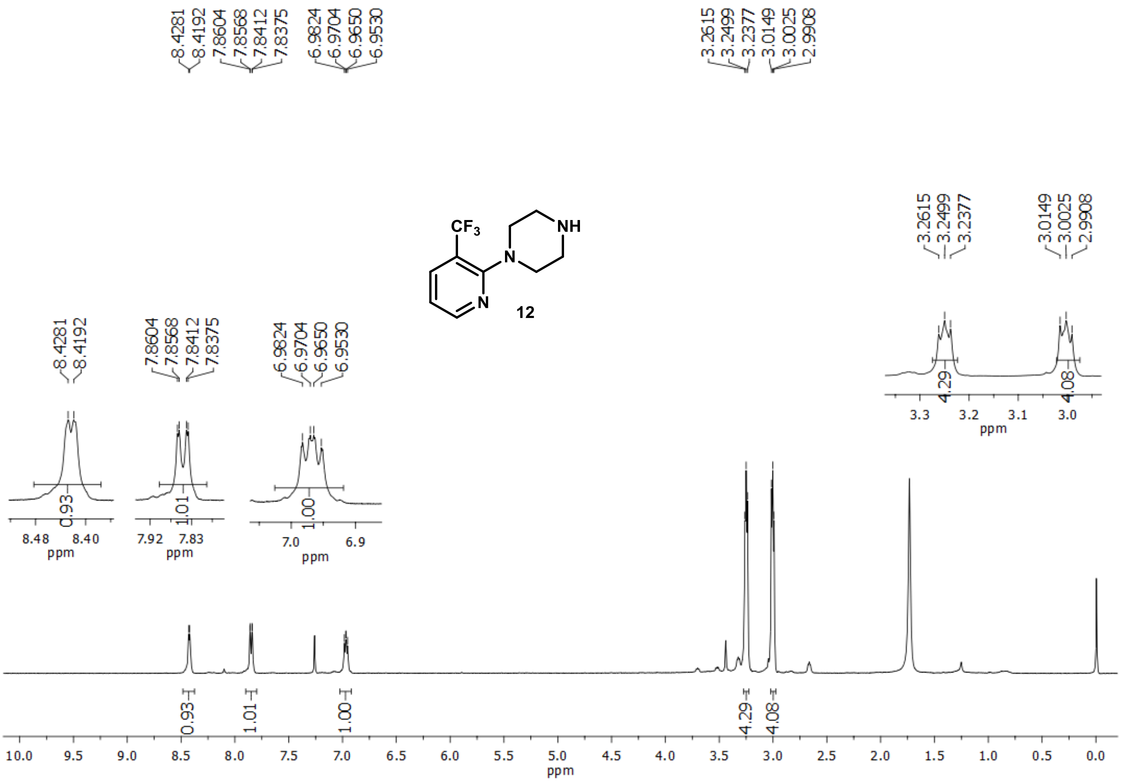

Figure S3 – ^{1}H NMR spectrum of **12** (400 MHz, $CDCl_3$)

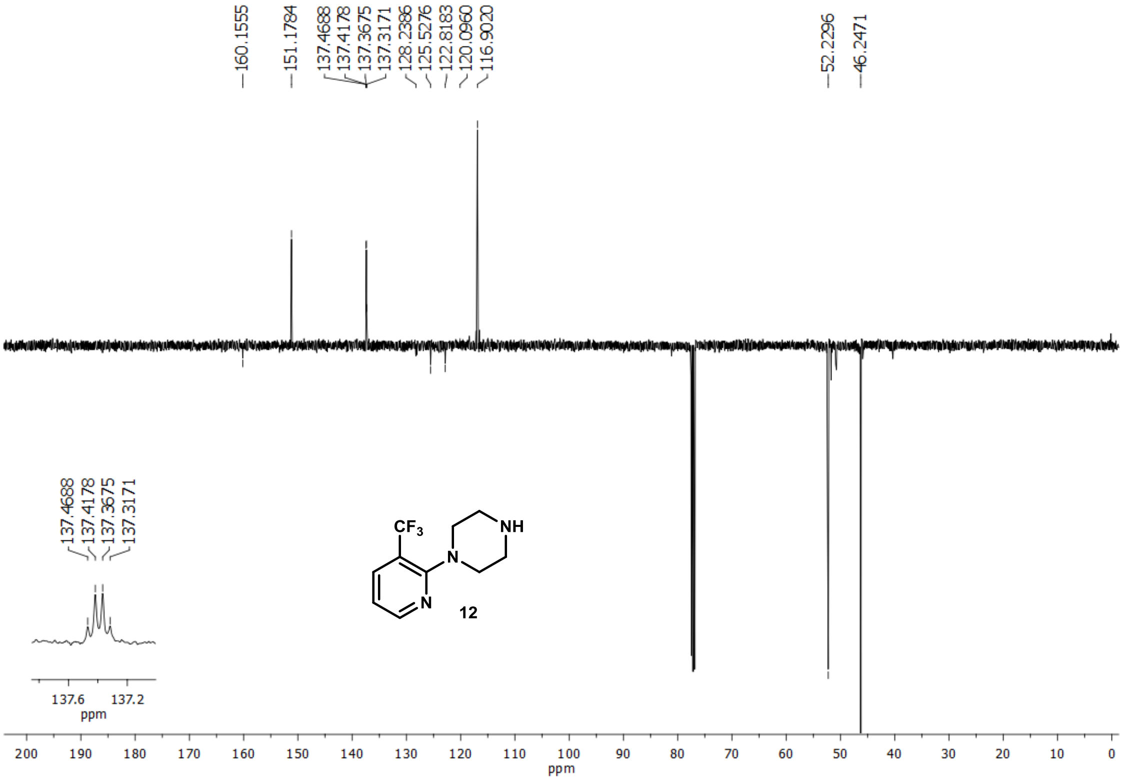

Figure S4 – ^{13}C APT NMR spectrum of **12** (101 MHz, $CDCl_3$)

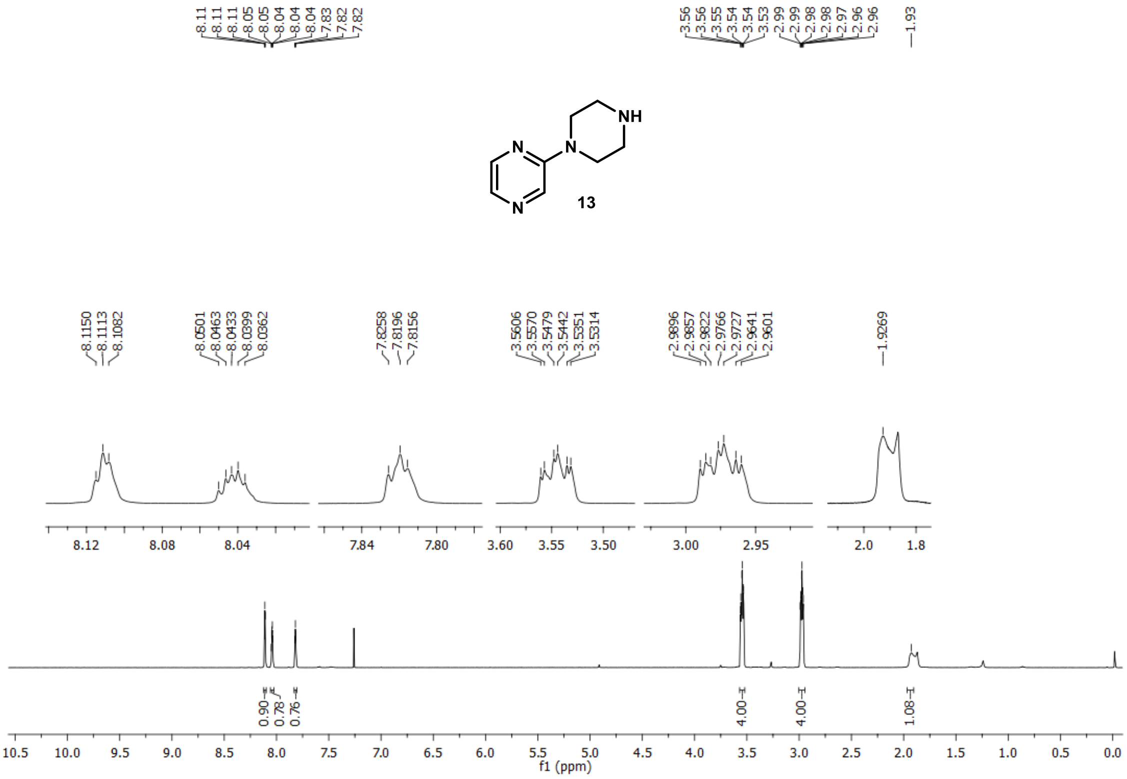

Figure S5 – ^{1}H NMR spectrum of **13** (400 MHz, $CDCl_3$)

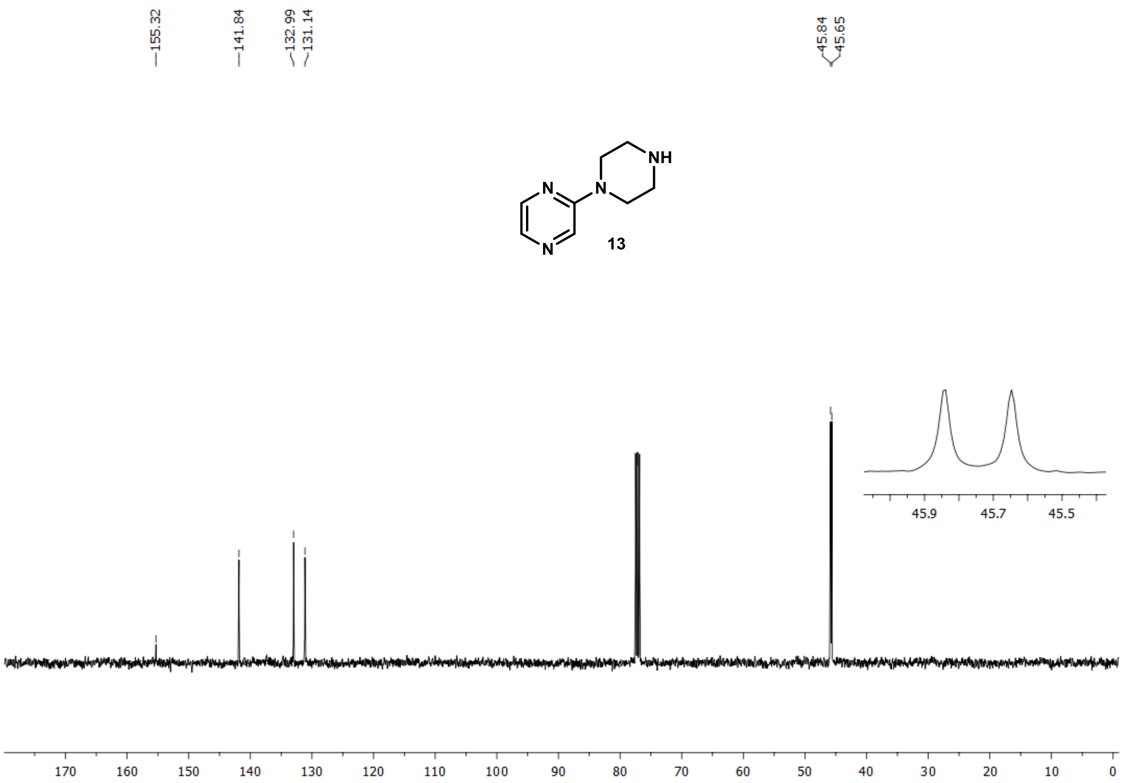

Figure S6 – ^{13}C NMR spectrum of **13** (101 MHz, $CDCl_3$)

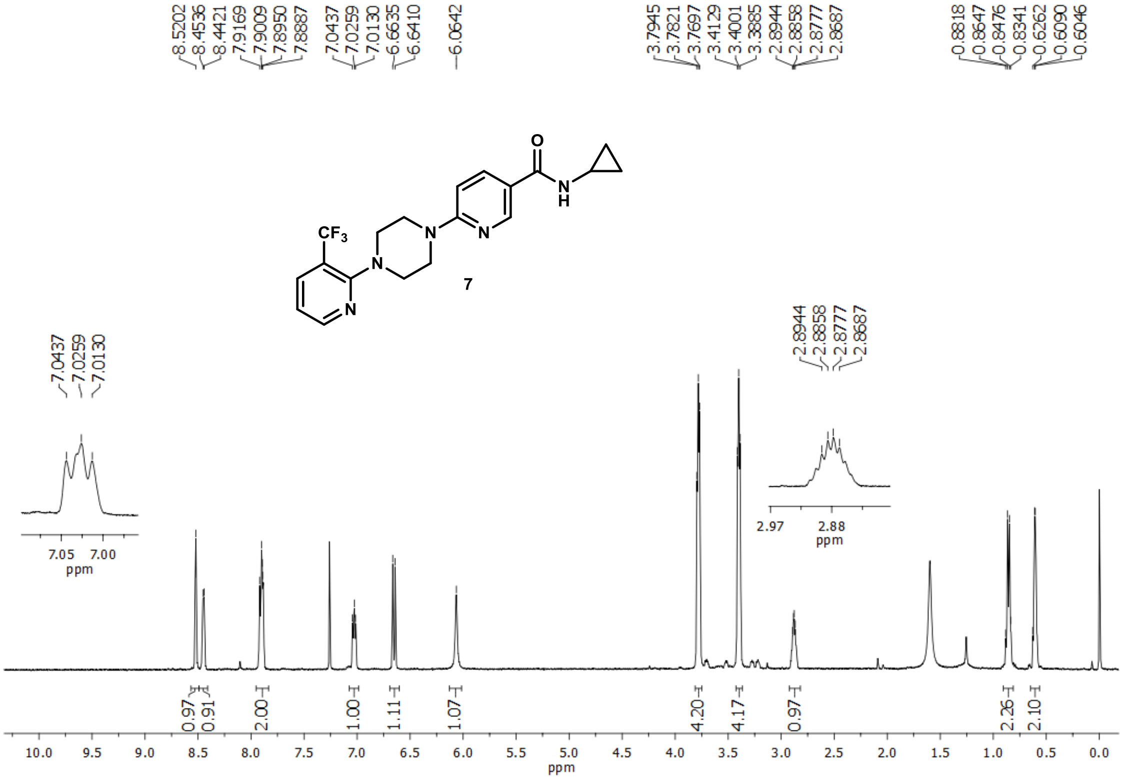

Figure S7 – ^{1}H NMR spectrum of **7** (400 MHz, $CDCl_3$)

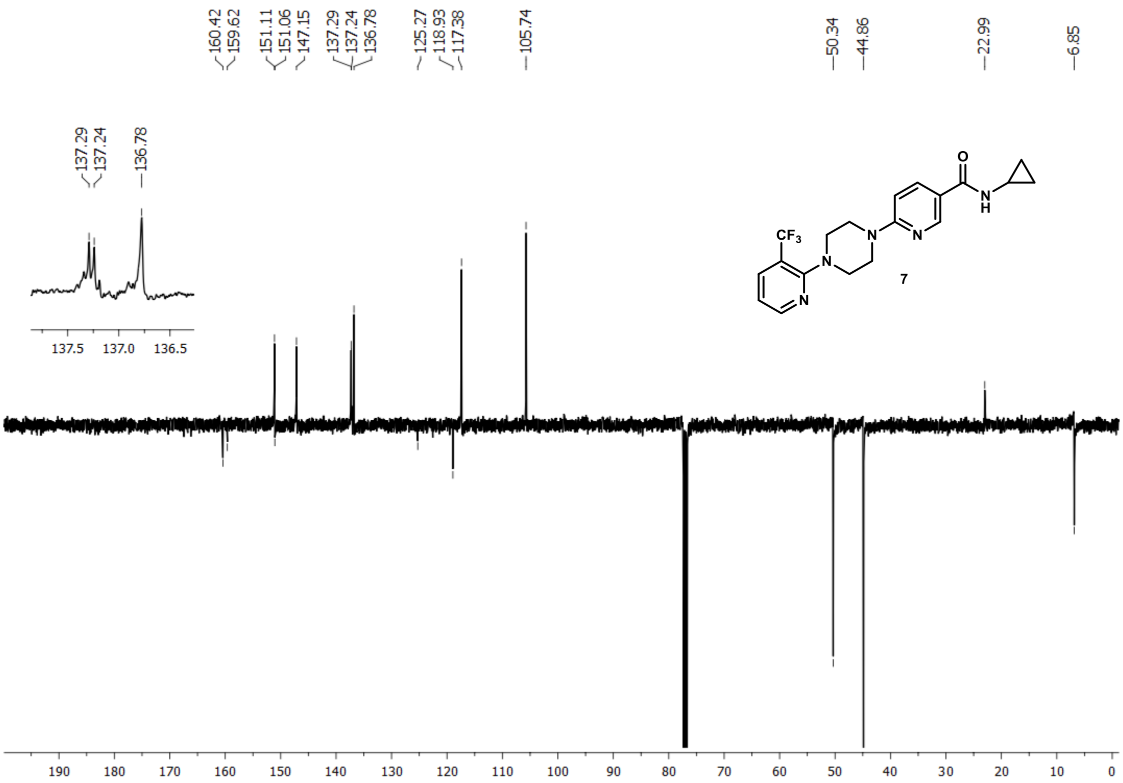

Figure S8 – ^{13}C APT NMR spectrum of **7** (101 MHz, $CDCl_3$)

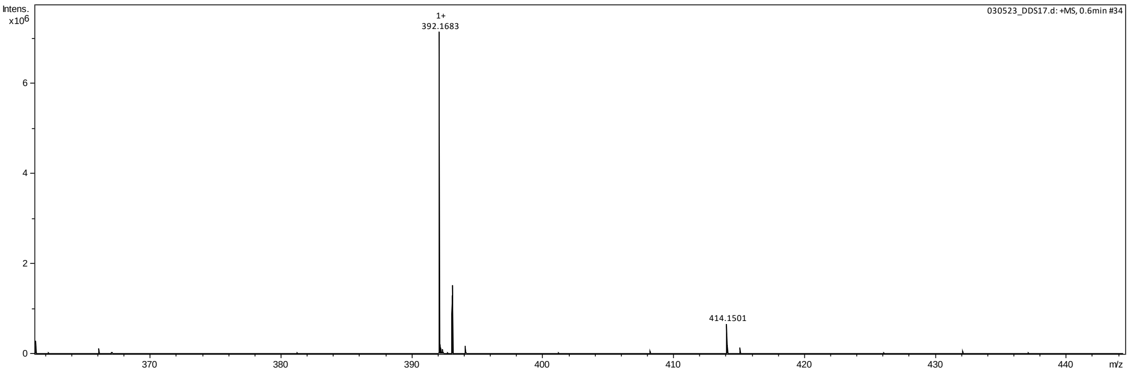

Figure S9 – HRMS (ESI +) spectrum of **7**

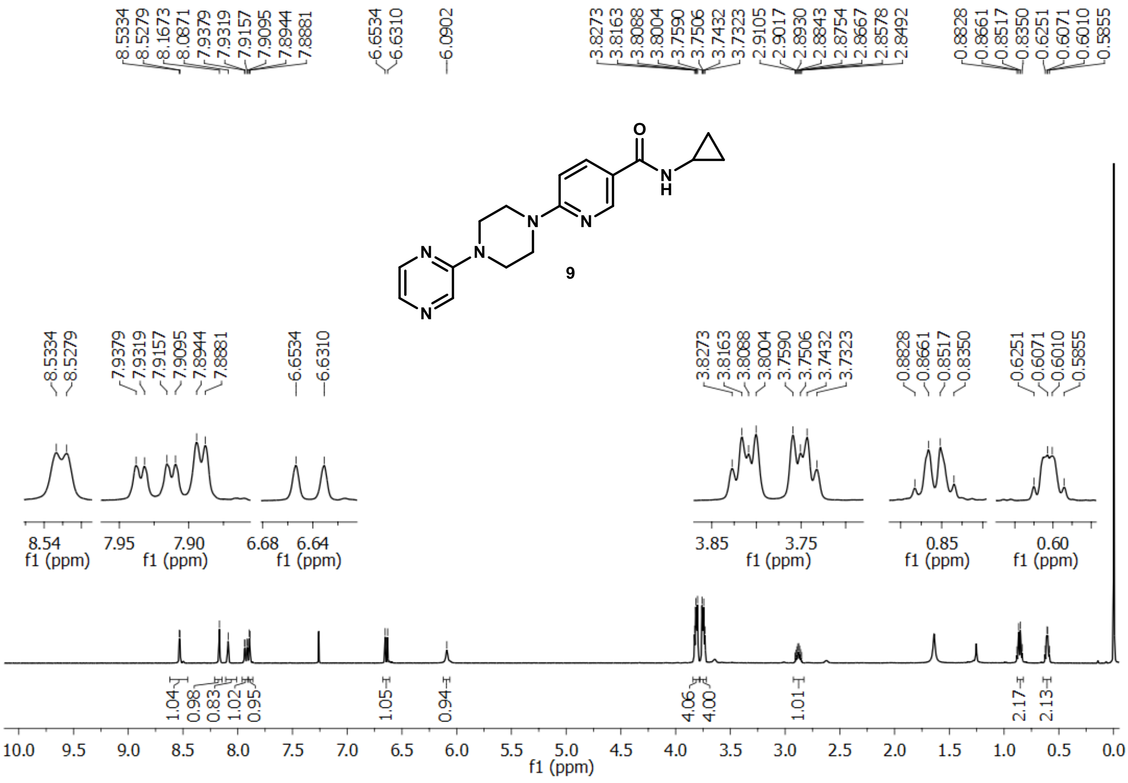

Figure S10 – ^{1}H NMR spectrum of **9** (400 MHz, $CDCl_3$)

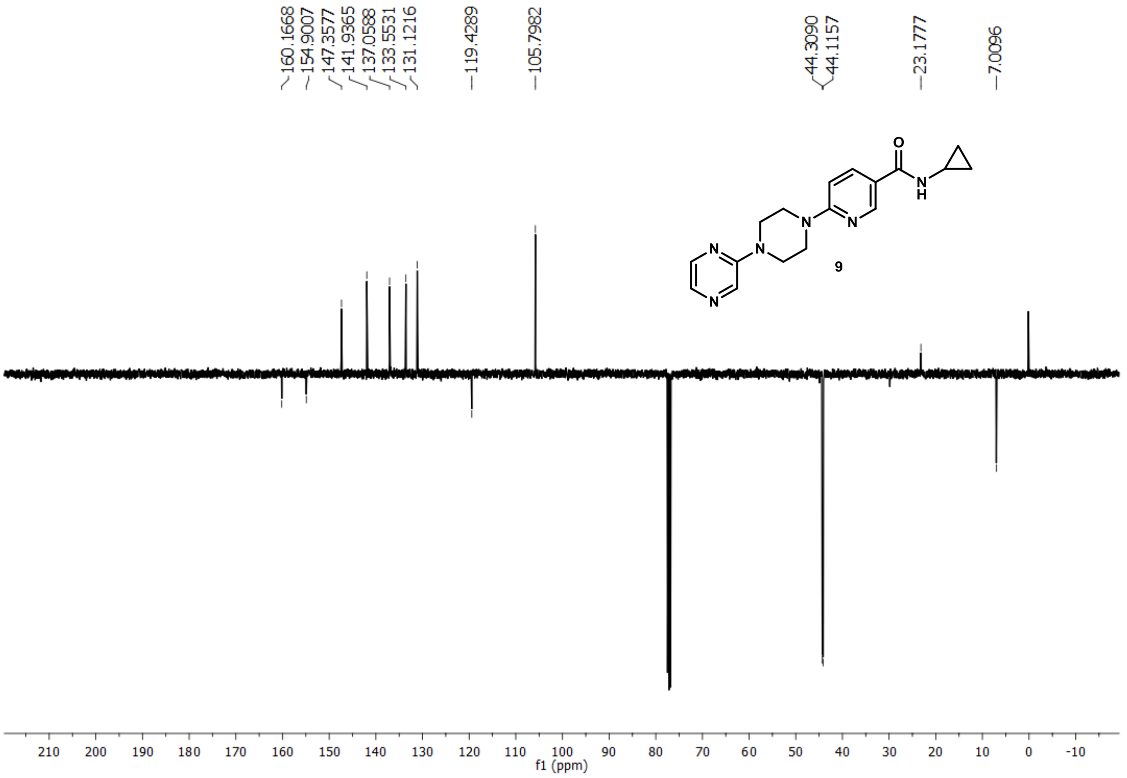

Figure S11 – ^{13}C APT NMR spectrum of **9** (101 MHz, $CDCl_3$)

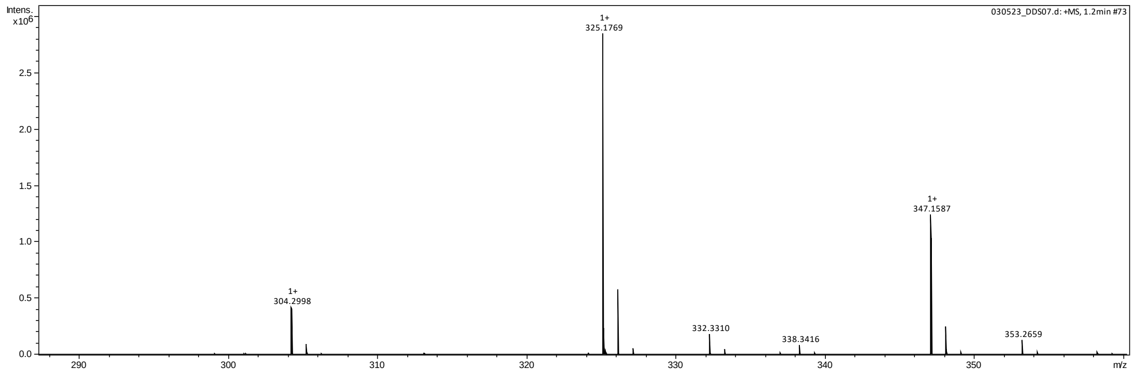

Figure S12 – HRMS (ESI +) spectrum of **9**

2. Purity and HPLC Analysis of compounds 7 and 9

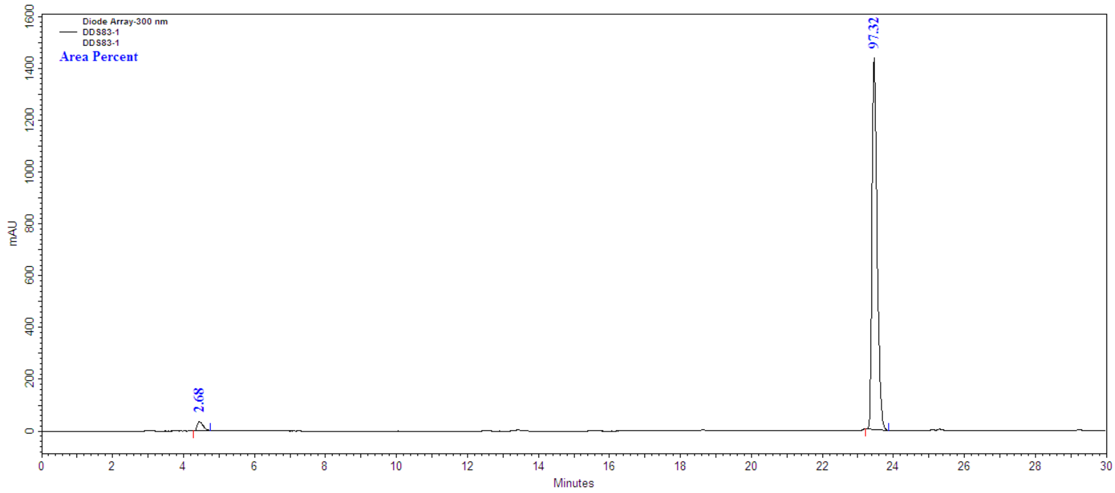

Figure S13 – Analytical HPLC chromatogram of compound **7.**

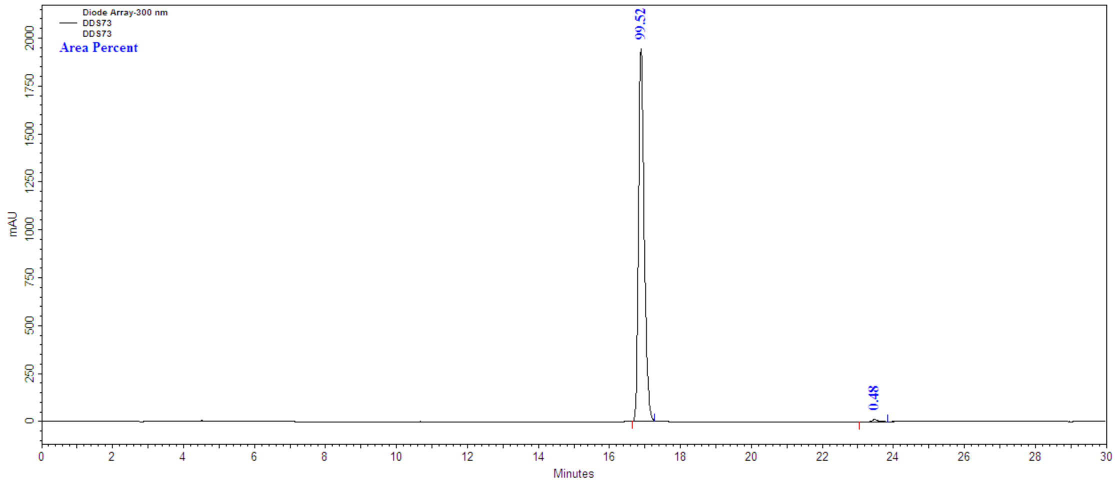

Figure S14 – Analytical HPLC chromatogram of compound **9.**
